# Metabolic syndrome in children and adolescents: definitions, epidemiology, pathophysiology, interventions, and challenges

**DOI:** 10.3389/fendo.2025.1512642

**Published:** 2025-06-11

**Authors:** Baoquan Zhang, Huiying Shi, Wenhong Cai, Bin Yang, Wenlong Xiu

**Affiliations:** Neonatology Department, Fujian Maternity and Child Health Hospital College of Clinical Medicine for Obstetrics & Gynecology and Pediatrics, Fujian Medical University, Fuzhou, China

**Keywords:** metabolic syndrome, children, adolescents, diabetes mellitus, obesity, cardiovascular diseases, epidemiology

## Abstract

Metabolic syndrome (MetS) is a group of cardiometabolic risk factors with high prevalence in the adult population. To date, there is no consensus on the definition for MetS in children and adolescents despite the presence of well-established diagnostic criteria in adults. The etiology of MetS is associated with a complex interaction between genetic susceptibility and environmental factors, in which the modifiable environmental risk factors are considered to play an important role in this process. MetS is significantly associated with an increased risk of diabetes mellitus and cardiovascular diseases (CVDs). Thus, it is necessary to pay attention to the prevention of MetS in childhood and adulthood. Given the current epidemic of obesity in children and adolescents, there is an urgent need to provide adequate guidelines for the definition, screening, and treatment strategies of MetS in younger patients. In this narrative review, we provide some diagnostic criteria and epidemiological studies and highlight the pathogenesis and management of MetS.

## Introduction

Metabolic syndrome (MetS) is a complex cluster of metabolic disorders characterized by disruptions in the metabolism of proteins, fats, and carbohydrates (1). It primarily includes central obesity, dyslipidemia, hypertension, and insulin resistance (IR) (2). In the past decades, MetS has been extensively studied in adult populations (3), however, our understanding of MetS in children and adolescents is still limited. Several large epidemiological cohort studies have demonstrated an association between MetS and cardiovascular outcomes in adults (4). These findings from adult studies, coupled with the rising prevalence of overweight among children and adolescents, have reignited interest in studying MetS in younger populations (5). As obesity-related MetS in childhood may persist into adulthood and is associated with cardiometabolic and psychosocial comorbidities, as well as premature death (6).

Patients with obesity usually present accumulation of free fatty acids (FFAs) in liver, adipocytes, skeletal muscle, and pancreas (7), which causes lipotoxicity in pancreatic β-cells and inhibition of insulin signaling in the liver and muscles, along with the eventual occurrence of IR (8). These patients show an increased risk of MetS and cardiovascular complications due to production of very low-density lipoprotein (VLDL) (9). Therefore, measurements with an aim to reduce the concentrations of cardiometabolic risk factors in children and adolescents can reduce the global burden of cardiovascular disease (CVD). This emphasizes the importance of MetS prevention in childhood. In this narrative review, we summarized the diagnostic criteria, epidemiology, pathophysiology, and treatment strategies of MetS in children and adolescents.

## Definition of MetS

MetS is defined by a series of physiological, biochemical, clinical, and metabolic factors. In 1988, Gerald Reaven first used the term “Syndrome X” to describe a specific cluster of cardiometabolic risk factors (10), and then gave rise to the concept of MetS. Since then, this cluster of risk factors was represented by various names, including “Deadly Quartet” (11), “IR Syndrome” (12) and “Metabolic Abnormality Syndrome” or “Diabetes.” In 2001, the National Cholesterol Education Program (NCEP) Adult Treatment Panel III (ATPIII) coined the term “MetS” and provided its definition (13). Subsequently, numerous organizations, including the World Health Organization (WHO) (14), the International Diabetes Federation (IDF), and the National Heart, Lung, and Blood Institute (NHLBI), issued their definitions. These definitions generally include aspects such as central obesity, hyperglycemia, hypercholesterolemia, low high-density lipoprotein cholesterol (HDL-C), and elevated blood pressure (BP) (**Table 1**). Although there are some similarities, these definitions differ in their threshold values for biochemical parameters and the targeted populations.

**Table 1:** Adult definitions.

	WHO (1999)	NCEP ATP III (2001)	IDF (2005)
Number of items for diagnostic inclusion	Dysglycemia, plus 2 or more of the other 4 criteria	Any three of the five criteria below	Obesity, plus two of the four criteria below
Dysglycemia	IR, impaired glucose regulation or diabetes	FPG ≥ 6.1 mmol l^-1^/100 mg dl^-1^ or known T2DM	FPG ≥ 6.1 mmol l^-1^/100 mg dl^-1^ or known T2DM
Central obesity	Waist to hip ratio > 0.90 (M), > 0.85 (F); and/or BMI > 30 kg m^-2^	WC ≥102 cm (M), ≥ 88 cm (F)	WC ≥ 94 cm (M), ≥80 cm (F)
Dyslipidemia	TG ≥1.7 mmol l^-1^; and/or low HDL-C < 0.9 mmol l^-1^ (M), < 1.0 mmol l^-1^ (F)	TG ≥1.7 mmol l^-1^; HDL-C < 1.04 mmol l^-1^ (M), < 1.3 mmol l^-1^ (F)	TG ≥1.7 mmol l^-1^; HDL-C < 1.04 mmol l^-1^ (M), < 1.3 mmol l^-1^ (F)
Hypertension	BP ≥ 140/90 mmHg	BP ≥ 130/85 mmHg	BP ≥ 130/85 mmHg
Microalbuminuria	urinary albumin excretion rate ≥ 20 µg min^-1^ or albumin:creatinine ratio ≥ 30 mg g^-1^	Not used for diagnosis	Not used for diagnosis

To the best of our knowledge, the definition of adult MetS cannot be simply used in children and adolescents, as the body size and proportions show a significant change with age. There are also remarkable changes in the fat distribution, insulin sensitivity of muscle and liver, and insulin release between adolescents and adults (15). Even in children and adolescents, there is no consensus on the definition of MetS. Its diagnosis requires assessment of waist circumference (WC), BP, lipids, and glucose (**Table 2**).

**Table 2:** Summary of the definitions of the MetS in children and adolescents.

	IDF (2007)		CPS/CMA (2012)		Cook (2003)	Weiss (2004)	de Ferranti (2006)	IDEFICS (2014)
Age Diagnostic criteria	6 to <10 years MetS cannot be diagnosed at this age.	10 to <16 years Central obesity and ≥ 2 components	6 to <10 years MetS cannot be diagnosed at this age.	10 to <16 years Central obesity and ≥ 2 components	12 to 19 years ≥ 3 components	4 to 20 years ≥ 3 components	12 to 19 years ≥ 3 components	2 to 11 years ≥ 3 components
Central obesity	WC≥P_90_	WC ≥ P_90_	Without cut-off definition for MetS diagnosis	WC ≥ P_90_	WC ≥ P_90_	BMI z-score ≥ 2.0	WC ≥ P_75_	WC ≥ P_90_
Dysglycemia	Without cut-off definition for MetS diagnosis	FPG ≥ 5.6 mmol l^-1^ or known T2DM	Without cut-off definition for MetS diagnosis	FPG ≥ 5.6 mmol l^-1^; or 7.8≤ OGTT 2h glucose < 11.1 mmol l^-1^ or T2DM	FPG ≥ 6.1 mmol l^-1^	OGTT 2h glucose ≥ 7.8 mmol l^-1^	FPG ≥ 6.1 mmol l^-1^	Insulin ≥P_90_ or FPG ≥ P_90_
Dyslipidemia	Without cut-off definition for MetS diagnosis	TG ≥ 1.7 mmol l^-1^; HDL-C < 1.03 mmol l^-1^	Without cut-off definition for MetS diagnosis	TG ≥ 1.7 mmol l^-1^; HDL-C < 1.03 mmol l^-1^ or non-HDC-C ≥ 3.76 mmol l^-1^	TG ≥ 1.24 mmol l^-1^; HDL-C < 1.03 mmol l^-1^	TG > P_95_; HDC-C< P_5_	TG ≥ 1.1 mmol l^-1^; HDL-C < 1.3 mmol l^-1^ (M), < 1.17 mmol l^-1^ (F)	TG ≥ P_90_; HDL-C ≤ P_10_
Hypertension	Without cut-off definition for MetS diagnosis	BP ≥ 130/85 mmHg	Without cut-off definition for MetS diagnosis	SBP or DBP ≥ P_95_	SBP or DBP ≥ P_90_	SBP or DBP ≥ P_95_	SBP or DBP ≥ P_90_	SBP or DBP ≥ P_90_

BP, Blood pressure; BMI, Body mass index; CMA, Chinese Medical Association; CPS, Chinese Pediatric Society; DBP, Diastolic blood pressure; FPG, Fasting plasma glucose; F, Female; HDL-C, High-density lipoprotein cholesterol; IDEFICS, Identification and prevention of dietary- and lifestyle-induced health effects in children and infants; IDF, International Diabetes Federation; IR, Insulin resistance; M, Male; MetS, Metabolic syndrome; OGTT, Oral glucose tolerance test; SBP, Systolic blood pressure; TG, Triglyceride; T2DM, Type 2 diabetes mellitus; WC, Waist circumference; P_90_, 90th percentile.

In 2003, Cook et al. assessed adolescents aged 12–19 years based on the NCEP/ATP-III definition, using modified criteria that included a WC above the 90th percentile (P_90_), BP above the limits set by the National Blood Pressure Education Program, lipid levels exceeding the pediatric thresholds set by the NCEP, and glucose levels above adult values (16). In 2004, body mass index (BMI) was adopted as a basis by Weiss et al. even though abdominal obesity may vary by race (17). Two years later, de Ferranti et al. proposed a definition similar to Cook’s but with lower thresholds for WC and lipid levels, which may result in a higher prevalence of MetS (18). Shortly thereafter, the IDF introduced a new definition based on its adult criteria. They categorized children into different age groups. For children aged 6–10 years, metabolic and BP variables were not well-defined, and only WC was evaluated. For children aged 10 years or more, MetS could be diagnosed with abdominal obesity and the presence of two or more clinical features (e.g. elevated TGs, low HDL-C, hypertension, or elevated glucose). For children aged 16 years or more, the IDF adult criteria were used (19). In this new definition, WC percentiles were used instead of absolute values to account for differences in child development and racial background. In 2014, European researchers proposed a definition of MetS for prepubertal children (ages 2-11) in the identification and prevention of dietary- and lifestyle-induced health effects in children and infants (IDEFICS) study. This definition addressed the limitations of previous pediatric definitions and the need for early diagnosis (20). The criteria included obesity (WC ≥ P_90_), TGs ≥ P_90_, HDL-C ≤ 10th percentile [P_10_], BP (systolic blood pressure [SBP] or diastolic blood pressure [DBP] ≥ P_90_), and glucose (insulin ≥ P_90_ or fasting plasma glucose [FPG] ≥ P_90_). Percentiles were used as references, better compensating for differences in child development and racial background.

In 2012, China adopted a definition of MetS for children and adolescents based on the IDF and ATP III criteria, established through consensus by experts from the Chinese Pediatric Society (CPS) of the Chinese Medical Association (CMA) (21). For children aged ≥10 years, central obesity is a prerequisite for MetS, defined as a WC ≥ P_90_ for age and sex, along with at least two of the following factors: hyperglycemia, hypertension, low HDL-C or high non-HDL-C, and hypertriglyceridemia. For children aged 6–10 years, whose physiological characteristics change rapidly, the diagnosis of MetS is still a challenge, and multiple CVD risk factors (e.g. obesity, hypertension, lipid metabolism disorders, and hyperglycemia) should be noted. Early intervention is recommended for children in this age group who exhibit multiple metabolic abnormalities. The definition proposed by CPS/CMA is similar to the IDF adolescent version but differs in certain thresholds and assessment items. The method for determining central obesity is different from the IDF’s obesity rate assessed by WC ≥ P_90_. Instead, it uses the waist-to-height ratio (WHtR), with a threshold of 0.48 for boys and 0.46 for girls (22).

Overall, the definition proposed by the IDF is the most effective and widely used in clinical practice. Due to significant variations in metabolic and physiological characteristics based on age and sex during the growth and development of children and adolescents, as well as notable differences in dietary habits and lifestyles across countries and regions, there is no consistent definition of MetS in children. We then identify common mechanisms to facilitate the establishment of a comprehensive and accurate definition and diagnostic criteria for MetS in children and adolescents.

## Epidemiology

It is estimated that approximately 39% of the global population is facing challenges of overweight, and the prevalence of overweight conditions is gradually increasing among children and adolescents (23). MetS is a complex disease that has been extensively studied in the adult population, but information on the prevalence in pediatric population is still limited (3). The epidemiology of MetS varies greatly between nations, and the prevalence is mainly associated with the diagnostic criteria, obesity rates, and race (**Table 3**) (24–31).

**Table 3:** Summary of the prevalence of the MetS in children and adolescents.

Characteristics	Diagnostic criteria	Published	Population	Age	MetS prevalence in the total	MetS prevalence in obese	MetS prevalence in boy	MetS prevalence in girl
Cruz et al. (24)	NCEP ATP III	2004	Overweight Hispanic children (mean BMI 97th percentile; n = 126)	8-13	30.0%	30.0%	ND	ND
Agirbasli et al. (25)	NCEP ATP III	2006	Turkish students (n=1385)	10-17	2.2%	21.0%	3.2%	1.0%
Barzin et al. (26)	Cook	2018	Non-obese children in Tehran (n=1033)	7-11	6.7%	ND	5.5%	7.7%
Ávila-Curiel et al. (27)	Cook	2018	Mexico State public school students (n=1017)	6-12	43.9%	54.6%	43.5%	44.3%
Jankowska et al. (28)	IDF	2021	Caucasian obese children of Gdańsk, Poland (n=591)	10-12	12.9%	12.9%	14.6%	10.9%
Xu et al. (29)	IDF	2012	Students from 6 provincial capitals in China (n=2647)	10-11	0.8%	3.5%	1.0%	0.6%
Leone et al. (30)	IDF and IDEFICS	2020	Caucasian obese children and adolescents(n=229)	7-20	19.9%	ND	ND	ND
Ramirez-Velez et al. (31)	de Ferranti	2018	Colombian children and young people (n=1047)	9-12	ND	ND	12.9%	14.6%

MetS, Metabolic syndrome; ND, Not determined.

To date, there is still no consensus on the diagnosis of MetS in children, and the cutoff values are in a huge difference that yields to various prevalence (32). Take the IDF criteria as an example, the Spanish study found that the prevalence of MetS varied from 2.5% in adolescents with a mean age of 13 years to 19.6% in children and adolescents aged 5–19 years (33). However, the results are not consistent when using different diagnostic criteria to the same population. Based on the NCEP/ATP III diagnostic criteria, Peña-Espinoza et al. found that the prevalence of MetS in children aged 9–12 years was 21.1%, 15.5% using the IDF criteria, 13.8% using the Cook criteria, and 45.9% using the De Ferranti criteria (34). Serrano et al. reported a prevalence of 9.5% for MetS in children aged 6–10 years using the NCEP/ATP III criteria and 8% using the IDF criteria (35).

The overall prevalence of MetS in children is relatively low, while that in overweight adolescents shows a 4–8 fold increase (36). The prevalence of MetS in European pediatric populations ranges from 1.44% to 55.8% (37). In a previous study, the global prevalence of MetS in 2020 was estimated at 2.8% in children and 4.8% in adolescents (38). A comprehensive review of 85 studies mostly using IDF, ATP III, and WHO criteria concluded that the median prevalence of MetS in the general population was 3.3% (ranging from 0% to 19.2%), 11.9% in overweight children (ranging from 2.8% to 29.3%), and 29.2% in obese populations (ranging from 10% to 66%) (39). In a systematic review, Sharma et al. reported that the prevalence of MetS in children and adolescents was 3.4% in normal-weight groups and 29% in obese groups (36). In 2012, China adopted the NCEP-ATP III diagnostic criteria, adjusted for age- and sex-specific WC and BP. In a study performed in Jiangsu Province, the prevalence of MetS was 5.1% among children and adolescents aged 7–17 years, 5.9% among those aged 13–17 years, and the prevalence of MetS in obese populations showed 40.2-fold increase compared to normal-weight peers (40). This highlights that the obesity rate within a study population is directly related to the prevalence of MetS.

Generally, ethnicity has been reported to be closely associated with the prevalence of MetS. Globally, the prevalence of MetS was higher in the following regions or ethnic groups. In the Middle East especially the Iran showed a prevalence of 7.6% according to IDF standards (41), 9.8% in the United Arab Emirates (38), and 20.6% in the Saudi Arabia based on to de Ferranti’s standards (42). In Europe, the prevalence of MetS in Spain was 9.9% (38). In North America, the United States showed a prevalence of 5.4% according to IDF standards (43), 10.1% according to Ford et al. and 12.3% in Mexico (38, 44). In South America, Chile showed a prevalence of 9.5% according to IDF definition (38, 45). In the United States, Miller et al. reported that the prevalence of MetS varied across different ethnic groups, with Hispanic adolescents showed the highest rate of 14.6%, followed by non-Hispanic whites (9.8%) and non-Hispanic blacks (5.2%) (44). This was consistent with the latest report from the US NHANES population (46). Some studies have found that despite the high obesity rate among African American adolescents (23.6%), their prevalence of MetS is relatively low (47). These findings suggest that the impact of obesity on MetS may vary by ethnicity. In summary, there are differences in the prevalence of MetS among different ethnic groups, but there is no consistent pattern.

## Risk factors and pathophysiology

### Genetic factors

**Figure 1:**
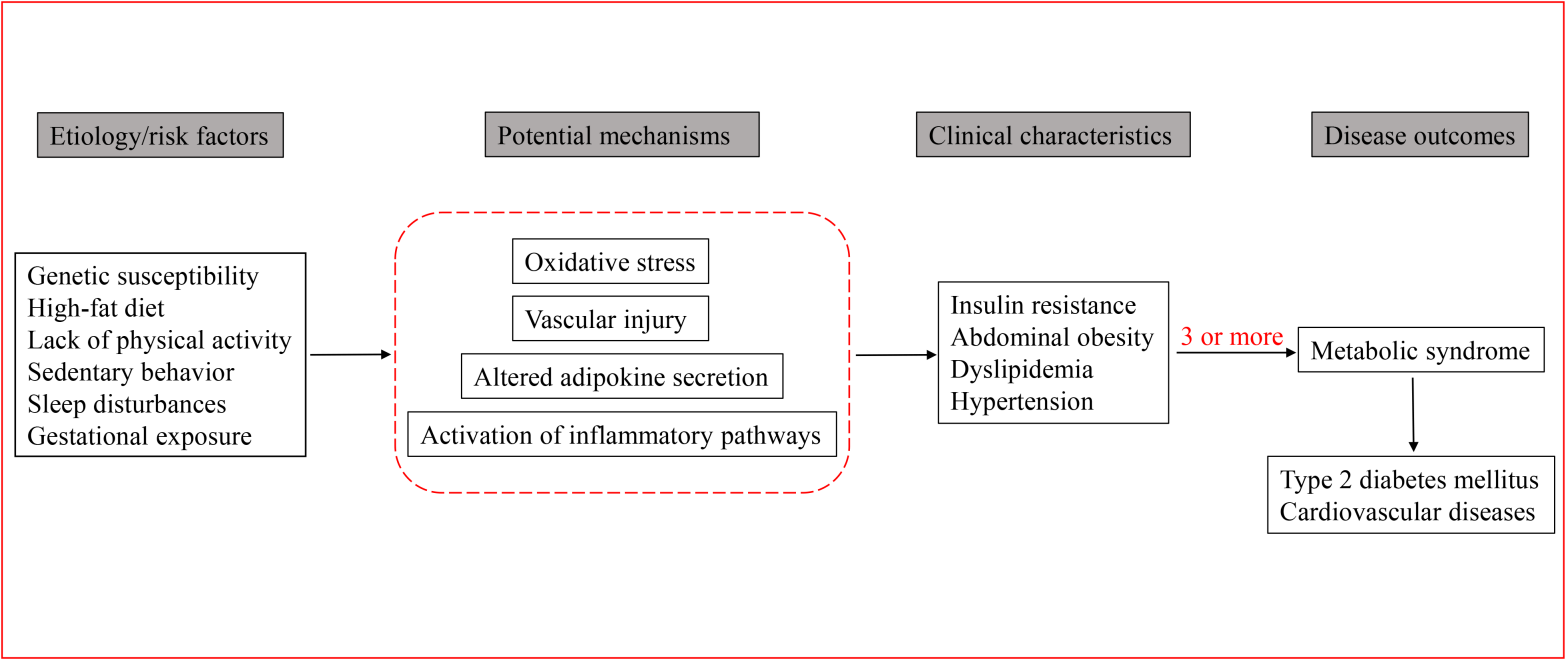
The MetS developmental process: a path diagram from etiology to disease outcome.

MetS is the result of a complex interaction between genetic and environmental factors (48). **Figure 1** depicts the MetS developmental process, involving genetic factors, oxidative stress, and inflammation pathways. Currently, great attention has been paid to the association between genes and individual components of MetS in children and adolescents, such as the relationship between certain genes and obesity, lipid levels, or IR.

The Fat Mass and Obesity-Associated (*FTO*) gene, located on chromosome 16, plays a critical role in body weight regulation and energy balance (49). The A/A phenotype of the risk allele rs9939609 (T/A) is closely associated with the development of obesity, a correlation observed in children as well (50). Among the polymorphisms of *FTO*, rs9939609 is the most widely studied locus, and increasing evidence suggest that it plays a central role in the development of MetS (51, 52). Almén et al. studied the genome-wide DNA methylation profiles of prepubescent girls with different variants of the rs9939609 polymorphism and identified 20 differentially methylated obesity-related loci (53). These findings suggest that increased *FTO* transcription in carriers of the A allele of rs9939609 may contribute to the higher risk of MetS. The latest study has updated the evidence, and some scholars have found that rs8050136 on the *FTO* is most strongly associated with MetS in children (52). However, the exact mechanisms by these single nucleotide polymorphisms (SNPs) increase the risk of MetS in children remain unclear. In a study, the author speculated that FTO variants could interfere with the methylation status of FTO target mRNAs and other non-coding RNAs, leading to an imbalance in energy intake and expenditure (54). Besides, the A allele of rs9939609 is associated with increased appetite and reduced satiety (55), resulting in increased energy intake in children. The leptin sensitivity showed reduction in the individuals with obesity, resulting in ineffective satiety responses and excessive hunger (56). Furthermore, energy expenditure may also involve in the association between *FTO* polymorphisms and MetS components. This helps to explain the fact that children carrying A allele are significantly associated with reduced physical activity.

Cholesteryl ester transfer protein (*CETP*) promotes the exchange of cholesteryl esters from HDL or LDL to TG-rich lipoproteins, resulting in reduced HDL-C concentration and generation of small-sized LDL particles (57). The *CETP* gene, located on chromosome 16 encoding the CETP protein, is reported to show a close link with the pathogenesis of MetS. (58). For instance, rs708272 was closely associated with increased HDL and reduced TG levels (52). In addition, a significant interaction was reported between the rs11774572 polymorphism and *CETP*-TaqIB (59), but the mechanism is still not well defined. This SNP is located between the GATA binding protein 4 (*GATA4*) and retinitis pigmentosa 1 (*RP1*) genes, which play a key role in cholesterol metabolism. *GATA4* encodes a transcription factor that mediates the transport of cholesterol and phytosterols and inhibits their abnormal accumulation (60). Variants in the *RP1* gene alter the lipoprotein phenotype by changing plasma TG and HDL-C concentrations, leading to hypertriglyceridemia (61). Therefore, the potential linkage disequilibrium between rs11774572 and functional mutations in these two genes may help to define the roles of *CETP* in MetS.

Furthermore, the rs662799 on the Apolipoprotein A5 (*APOA5*) gene is associated with high TG levels in both adults and children, as it could inhibit the activation of lipoprotein lipase (59). Similarly, a GWAS study in Korean population also revealed significant or suggestive loci for MetS in *APOA5* (62). In a study performed in Mexican population, the most commonly associated signal for TG was rs651821 in *APOA5*, followed by rs180326 in *BUD13* (63).

Studies have focused on the link between Caucasian and Asian adolescents and the *TCF7L2* in the pathogenesis of MetS (64). There is evidence that carriers of the *TCF7L2* rs7903146 risk allele have higher fasting insulin concentrations, impaired insulin sensitivity, and greater IR compared to CC homozygotes (65). *TCF7L2* gene polymorphisms increase the risk of T2DM by altering its gene expression, disrupting glucose homeostasis, impairing insulin secretion, and weakening insulin sensitivity (66). In addition, increased nut intake may reduce the risk of MetS in the T risk allele of *TCF7L2* rs7903146 and rs12255372 variants (67).

The prevalence of MetS in children and adolescents may be controlled by later intervention of environmental factors when genetic variation cannot be modified (68). There are indeed studies designed to investigate the association of genes with a single MetS disease, however, they did not take the fact that most genetic loci have pleiotropic effects on multiple MetS components into consideration. Therefore, it is appropriate to evaluate the effect of each SNP on MetS risk.

#### Early life exposures

Early life exposures, primarily maternal behaviors during pregnancy, may contribute to the early development of MetS. Susceptibility to MetS begins before birth, as obesity during pregnancy and associated gestational conditions (e.g. gestational diabetes, hypertension, and hyperlipidemia) increase the risk of obesity and metabolic disorders in offspring (69). Therefore, the pre-pregnancy and perinatal periods provide women and their offspring with a unique opportunity to modify short-term and long-term risks. A large number of observational studies have found that maternal obesity, hypertension, and hyperglycemia during pregnancy increase the risk of MetS in offspring (70).

Increased maternal glucose levels are associated with a higher incidence of obesity in newborns (71). When examining the gene expression profiles in placentas from women with gestational diabetes and those with normal glucose tolerance, researchers found an upregulation of genes related to lipid metabolism. This indicated that lipids might serve as a nutritional source contributing to increased neonatal obesity (72, 73). Consistently, Boney et al. reported that the offsprings of obese women had a higher likelihood of being obese at age 11, along with 2.0-fold increase in the risk of MetS (74).

The progenitor cells and adipocyte populations in subcutaneous adipose tissue have been formed in fetus, laying the foundation for an individual’s future fat distribution and metabolic health (75). This means that the “set point” for obesity has been determined *in utero*, and the intrauterine environment plays a crucial role in the development of MetS. Adverse intrauterine conditions such as obesity or diabetes accelerate the fat accumulation of fetal white adipose tissue (WAT) and disrupt its normal developmental trajectory (76). Furthermore, a high-sugar and high-fat condition promotes the stem cells to differentiate into adipocytes, and leads to a premature terminal differentiation process (77), which increases the susceptibility of offspring to obesity, limits the plasticity of WAT, and reduces its ability to adapt and regulate energy metabolism (78). In line with this, multiple rodent-based studies supported the long-term effects of maternal diet-induced obesity on the metabolic health of offspring. The offsprings have an increase in visceral adipose tissue volume, accompanied by a significant increase in the number and volume of fat cells, as well as significant IR (78).

It is worth noting that evidence has shown that epigenetics and prenatal programming might have influenced fetal/neonatal development, leading to MetS. The epigenome is dynamic and can change in response to factors such as nutrient availability and weight loss (79, 80). Intrauterine nutrition and environmental exposures may have permanently altered gene expression in offspring through epigenetic mechanisms, thereby changing the structure and function of cells and organs and resulting in metabolic abnormalities. This was well demonstrated in monozygotic twins, where offspring exhibited different DNA methylation and histone acetylation patterns (81). Epigenetic regulation of gene transcription is partly mediated through DNA methylation (82–84), which is particularly dynamic during embryogenesis. As embryonic development progresses, DNA methylation gradually increases, leading to differentiation and organ formation (85). This would promote the adipogenesis, while histone lysine methylation (H3K4) and acetylation (AcH3) can regulate adipocyte differentiation (86, 87). Higher pro-opiomelanocortin (POMC) methylation level in umbilical cord blood is associated with hyperinsulinemia in children, which may serve as a marker for future MetS (88). Epigenetic modifications such as DNA methylation and histone acetylation (89) are involved in the development and differentiation of pancreatic β-cells (90, 91). In the presence of poor intrauterine environment, pancreatic β-cells may undergo fetal developmental programming, resulting in a decrease in pancreatic β-cells number and/or dysfunction. All these may increase the risk of long-term metabolic complications in offspring.

### Environmental factors

**Figure 2:**
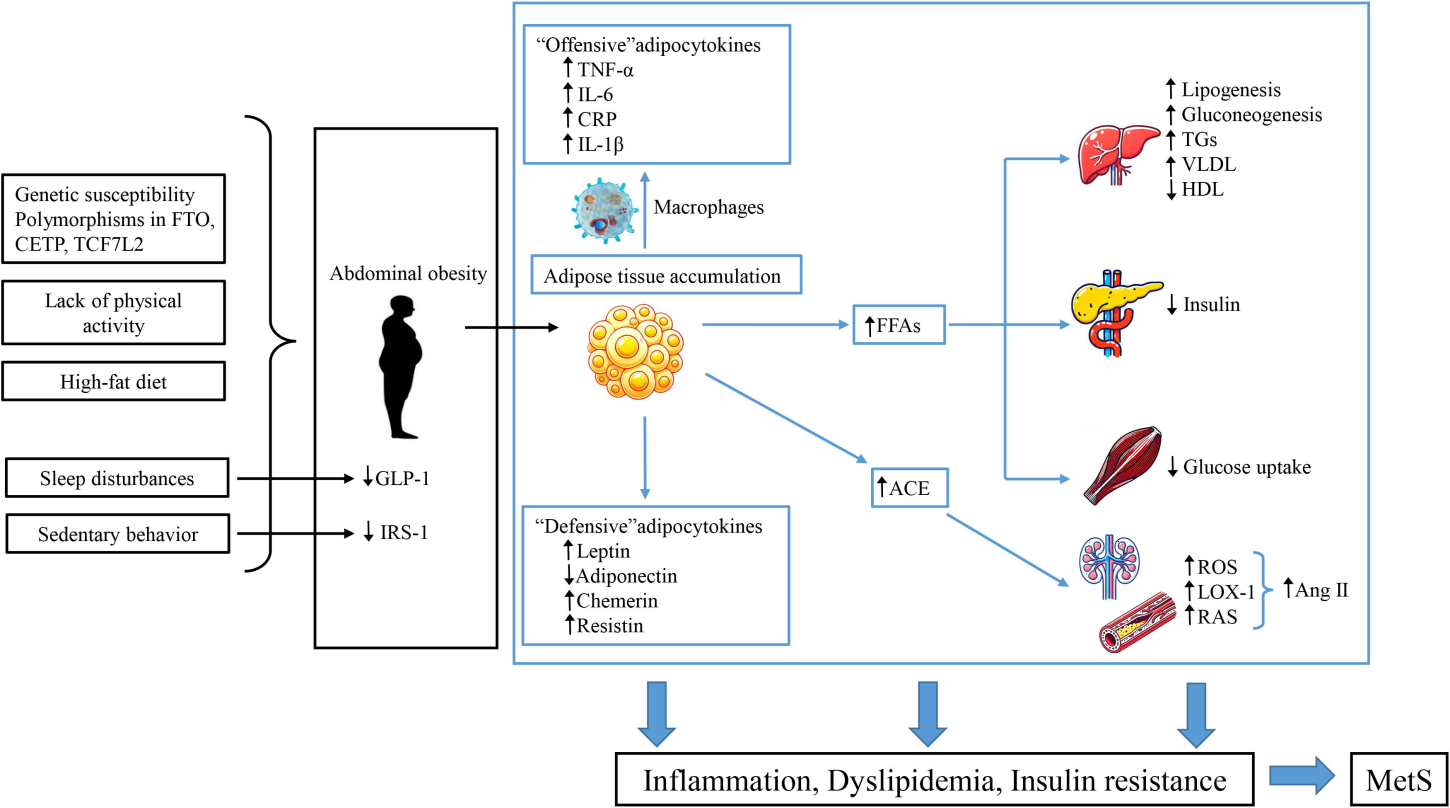
MetS pathogenesis in children and adolescents. This figure describes the potential risk factors and mechanisms underlying the pathophysiology of MetS in pediatric populations. Genetic susceptibility and unhealthy lifestyles contribute to central obesity, leading to an imbalance between “aggressive” adipokines produced by adipose tissue and macrophages and the dysfunction of “defensive” adipokines. This imbalance increases immune-inflammatory responses and promotes obesity-related metabolic disorders. The activation of adipose tissue leads to the production of angiotensin II (Ang II) peptides through angiotensin-converting enzyme (ACE), increasing OS and upregulating the expression of lectin-like oxidized low-density lipoprotein receptor-1 (LOX-1), inducing endothelial dysfunction. Additionally, the increased secretion of FFAs from adipose tissue leads to reduced sensitivity in insulin-responsive organs. This cascade ultimately results in IR, dyslipidemia, and hypertension, significantly increasing the risk of MetS, T2DM, and CVD.

SNPs are established at conception, while environmental factors such as diet and lifestyle influence the baseline during growth. This raises the question of which variable is the “cause” and which is the “moderator,” a common issue in studies of gene-environment interactions. Environmental factors that contribute to MetS in children include sedentary behavior, high-fat diets, insufficient sleep, and systemic or tissue inflammation. **Figure 2** depicts potential risk factors and mechanisms of MetS pathophysiology in children and adolescents. Epidemiological data indicates that sedentary lifestyles, lack of physical activity, and high-fat diets are key contributors to energy imbalance, closely linked to the prevalence of childhood obesity and metabolic disorders such as IR (92). Thus, we hypothesize that MetS begins with obesity but requires IR to progress to MetS in children, which was consistent with the hypothesis proposed by Weiss (17).

Several studies have suggested that there might be a link between high-fat diets, dietary fatty acids, and the risk of MetS (93, 94). The main components of the Western diet mainly include meat products, sugary drinks, junk food, refined grains, candy, and ultra-processed foods. A large amount of saturated fatty acids (SFA) and carbohydrates in these foods have been shown to be directly associated with an increased risk of MetS in children and adolescents (94–96). Dietary fatty acid is an important environmental factor, and excessive exposure has played a critical role in the development of MetS (97). Epidemiological and cohort studies have shown that SFA has an adverse effect on insulin sensitivity, promoting the development of diabetes (98). The Nurses’ Health Study found that higher SFA intake and a lower polyunsaturated to saturated fat (P:S) ratio were associated with an increased risk of CVD in women with T2DM (99). A cohort study indicated that the Western dietary pattern was linked to a high-risk metabolic cluster (100). In contrast, a Mediterranean dietary pattern or a higher Healthy Eating Index score, containing more grains, vegetables, fruits, milk, and meat/meat alternatives, was associated with the reduced prevalence of MetS (95). A recent study showed that dietary polyunsaturated fats (PUFA) modulated the genetic effect of TCF7L2 rs7903146 polymorphism on postprandial dyslipidemia (101). Therefore, adopting such a dietary pattern early in life could reduce the risk of MetS in children and adolescents.

There may be a possible threshold for the accumulation of fat in abdominal compartments and insulin-responsive tissues. Upon exceeding such threshold, the accumulation of lipids in these areas would be detrimental (102). Increased consumption of fructose and branched-chain amino acids, along with increased intracardiac lipid metabolites, lead to the serine phosphorylation of insulin receptor substrate-1 (IRS-1) (103). This results in defective skeletal muscle glucose uptake, along with decreased hepatic glycogen synthesis, and suppression of gluconeogenesis, which ultimately decrease hepatic insulin sensitivity (104). These changes further exacerbate IR and set the stage for obesity-related metabolic disorders.

Insulin sensitivity in adipose tissues decreases in children with obesity (105). Lipid deposition in muscle and liver also increases in those with obesity (106). Macrophage infiltration of subcutaneous and intraperitoneal fat depots induces local and systemic subclinical inflammation, which is closely related to poor lipid partitioning in obese adolescents (107). In addition, skeletal muscle precedes liver, followed by enterogenic circulating glucose to the liver. The liver responds to increased glucose flux by increasing *de novo* fat processes, which leads to increased intrahepatic fat, circulating fatty acids and TGs (108). Meanwhile, macrophage infiltration causes IR in adipose tissue, resulting in increase of lipolysis and decrease of lipogenesis (109). As a result, hepatic FFA flux increases, leading to enhanced TG synthesis and systemic hyperlipidemia (110).

MetS children are also accompanied by systemic and tissue inflammation, as evidenced by elevated levels of inflammatory cytokines, including interleukin-6 (IL-6), IL-18, and C-reactive protein (CRP) (111). Obesity and IR induce systemic oxidative stress (OS) that activates downstream inflammatory cascades, which accelerates the development of MetS (112). Excess OS was associated with increased adipogenesis and body fat mass, potentially linked to the overexpression of NADPH oxidase 4 (NOX4) and downregulation of AMP-activated protein kinase (AMPK) in adipocytes (113). Several key inflammatory markers have been reported to involve in obesity-induced inflammatory responses, including CRP, IL-6, and tumor necrosis factor-α (TNF-α) (114, 115). IL-6 plays a regulatory role in fat and glucose metabolism and can promote IR. In obesity, IL-6 is released from visceral adipocytes into the portal vein and directly acts on the liver to induce the production of CRP (116). In addition, IL-6 can increase the risk of thrombosis, and lead to atherosclerosis, inflammation and dysfunction of the vascular wall by activating the local renin-angiotensin system (RAS) pathway and promoting the expression of vascular cell adhesion molecules (117). TNF-α is mainly produced by macrophages in local adipose tissue, and its levels is proportional to the mass of adipose tissue and is closely related to IR (118). It weakens insulin metabolism through serine phosphorylation and inactivation of the insulin signaling pathway, and further exacerbates IR by increasing circulating FFA levels (119, 120). Obese children exhibit higher TNF-α levels than lean controls, which is associated with reduced LDL-C and increased TGs (121). In the future, more studies are required to investigate the feasibility of these inflammatory markers in the management of MetS and its complications.

Insufficient sleep profoundly impacts energy balance and overall metabolic health, thereby increasing the risk of obesity in adolescents. In an observational study involving 240 American adolescents, subjects slept for less than 8 hours on weekdays consumed a significantly higher percentage of calories from fat compared to those who slept for 8 hours or more (122). This indicated that insufficient sleep may lead to unhealthy eating habits and imbalance of metabolic health. Sleep disorders, including insufficient sleep, poor sleep quality, insomnia, and obstructive sleep apnea, lead to increased cortisol secretion by the adrenal cortex (123), which trigger increased calorie intake and excessive fat storage (124). Additionally, the severity of obstructive sleep apnea was correlated with higher cortisol levels, which can disrupt the normal response of glucagon-like peptide-1 (GLP-1) (110). Sleep disturbances can disrupt circadian rhythm, affecting GLP-1 production and glucose metabolism (125). In children, sleep reduction was closely associated with elevated fasting insulin concentrations, increased risk of IR, and decreased insulin sensitivity (126). These findings highlight the importance of adequate sleep in maintaining healthy metabolic function and preventing the occurrence of MetS in adolescents.

## Prevention and treatment

### Prevention

The previous section highlighted a range of risk factors for MetS. Beyond genetic factors, many of these risks are modifiable targets for preventive measures. From the perspective of childhood development, it appears essential to promote healthy nutrition and maintain normal body weight among adults of childbearing age, particularly considering the potential early exposure to these risks during pregnancy.

Breastfeeding has been confirmed as a protective factor against MetS. A systematic review involving 11 studies, 7 studies revealed a protective role of breastfeeding and MetS, particularly breastfeeding lasted for 6 months or longer (127). Besides, breastfeeding for more than 90 days significantly reduced the risk of MetS (128). Breastfeeding plays a protective role in preventing obesity in a dose-dependent manner (129). Additionally, breastfeeding for at least 3 months is associated with reduced risk of obesity, smaller WC, and fewer MetS-related complications in childhood and adolescence (130). Moreover, breastfeeding is linked to reduced risks of high cholesterol, hypertension, DM, glucose intolerance, and IR in adulthood (130, 131). Furthermore, breastfeeding can prevent prediabetes and MetS in offspring, regardless of GDM status, underscoring the importance of breastfeeding (132).

Breastfeeding helps to prevent obesity through the modulation of liver-hypothalamic communication and metabolism (133). Bioactive factors in breast milk, such as insulin, insulin-like growth factor-1 (IGF-1), and leptin, promote lean body weight and enhance appetite signaling (134, 135). This “positive programming” of nutrition and hormones may have profound implications for preventing MetS and related diseases. Other healthy lifestyle choices, including a balanced diet and regular exercise, are vital for avoiding MetS. Healthy dietary habits include consuming plenty of fruits, vegetables, and dietary fiber while reducing the intake of carbonated drinks and foods high in sugar, fat, and sodium (136). Taken together, the combination of breastfeeding and a healthy lifestyle will lay a solid foundation for improving the health of children and adolescents.

### Treatment

The progression from a healthy state to obesity, IR, and eventually to the development of MetS is consistently associated with an imbalance between energy intake and expenditure. By the time MetS manifests, this energy imbalance has often been present for an extended period. Therefore, the primary goal of intervention is to reduce energy intake while increasing energy expenditure. Unfortunately, it is challenging to motivate pediatric patients to change unhealthy lifestyles, as many children and adolescents have become accustomed to a comfortable yet suboptimal way of living. Effective methods include motivational psychological interviews to explore the motivations of adolescents, assessing their willingness to change (137). To the best of our knowledge, multidisciplinary and family-based lifestyle education program supplemented with psychological support is recommended for the treatment and prevention of MetS. Thus, psychological adjustment is the first step in treating MetS. Subsequently, developing individualized treatment plans based on patient characteristics can enhance adherence to therapeutic regimens among adolescents (137). For younger children, the emphasis was on combining breastfeeding with a balanced diet, along with adequate sleep.

### Lifestyle modifications

Basically, all successful treatment plans include interventions to reduce calorie intake and increase physical activity. According to recommendations from the American Academy of Pediatrics (AAP), the American Heart Association (AHA), and the WHO, the core of dietary intervention for children and adolescents is to increase the intake of vegetables and fruits while reducing the intake of sugar and saturated fat (138). The Chinese Society of Pediatrics recommends that children and adolescents should maintain food diversity in their diet, pay attention to the combination of meat and vegetables, and the combination of coarse and fine, and ensure the intake of fish, meat, milk, beans, and vegetables. The energy supply ratios of protein, fat, and carbohydrates are 12%-14%, 25%-30%, and 55%-65%, respectively (21). In terms of managing MetS, studies have shown that children and adolescents who adopt a Mediterranean diet, which mainly includes vegetables, fruits, fish, whole grains, beans, and olive oil, have significantly improved BMI, blood sugar, and blood lipid levels, especially in individuals with obesity or high risk of MetS (139). In addition, randomized controlled trial (RCT) have shown that reducing the intake of sugary drinks has a positive effect on weight management, thereby indirectly reducing the risk of MetS (140). For the blood lipid management, dietary adjustments, such as reducing the intake of simple carbohydrates (e.g. sugar and refined flour), can help control the phenotype of high TGs and low HDL-C. In contrast, increasing the intake of monounsaturated and PUFA can reduce TG levels and increase HDL-C levels (141). Additionally, whole grain intake is closely related to enhanced insulin sensitivity and reduced BMI in adolescents. In particular, dietary fiber intake can effectively reduce postprandial blood sugar fluctuations and has significant benefits for insulin sensitivity, obesity, and pancreatic function (142, 143). In terms of BP management, a meta-analysis of 10 RCTs showed that moderate reduction of salt intake can significantly reduce both SBP and DBP in children and adolescents (144). All these confirm that reasonable dietary adjustments are crucial to the long-term metabolic health of children and adolescents.

A lack of physical activity is associated with a higher risk of MetS, as indicated by a higher MetS z-score (145). Regular physical activity helps improve lipid profiles by reducing LDL and TG concentrations along with increasing of HDL (146). Exercise also enhances the clearance of plasma TGs and promotes the formation of HDL particles, leading to positive effects on lipid metabolism (147). Physical activity significantly improves insulin sensitivity, reduces IR and significantly lowers fasting insulin levels (148). Also, exercise offers benefits to vascular health, including improvement of endothelial function, reduction of SBP and DBP, decrease of abdominal fat, and triggering the anti-inflammatory responses (149). The most effective exercise interventions should last at least 12 weeks, with sessions conducted three or more times per week, with each lasting 60 minutes or longer (150, 151). Consequently, regular and appropriate physical activity is one of the key factors in preventing MetS.

As individually oriented obesity prevention strategies are not adequate in addressing the obesity epidemic, more attention has been paid on the shift towards environment- or community-based prevention measurement, which promotes healthier lifestyles by altering the social environment. In a perspective of public health, more attempts should be made on community health programs, along with school-based physical activity initiatives and promote healthy eating styles.

### Pharmacotherapy

Lifestyle modifications remain the primary approach for the prevention and treatment of childhood obesity and MetS, however, pharmacological and surgical interventions become necessary adjuncts in some extreme cases (152). Currently, there are no specific guidelines for pharmacological treatment of dyslipidemia related to MetS in children. For children and adolescents with severe lipid abnormalities, the use of statins to lower LDL-C has been shown to delay arterial damage. This treatment is typically recommended only for children aged 10 years and older (141). These children had fasting LDL-C levels persistently >190 mg/dL, or LDL-C levels >160 mg/dL along with a significant family history of early-onset CVD or two or more additional risk factors (153). In addition, GLP-1 analogs, such as liraglutide, have demonstrated long-term efficacy in treating obesity in adults. A small-scale trial of another GLP-1 medication, exenatide, has also shown potential efficacy and safety in treating severe obesity in adolescents (154). For children at high risk of IR, pharmacological treatment may be unavoidable. In a recent double-blind randomized trial, obese adolescents aged 12 to 19 years who were treated with metformin for 6 months showed significant improvements in glucose tolerance and fasting insulin levels (155, 156). In the setting of severe obesity, bariatric surgery is considered the most effective treatment, which can significantly reduce the weight of children and adolescents and improve related health risks, such as sleep apnea and T2DM. However, potential complications after surgery, such as malabsorption of vitamin D, calcium, and phosphorus, also need to be carefully considered (157). At present, drug treatment of metabolic syndrome in children in China is still in its infancy. and doctors should consider multiple factors before prescribing anti-obesity drugs, such as gender, age, drug contraindications, personal and family willingness, and cost. For children and adolescents with severe obesity or metabolic disorders, a comprehensive treatment strategy that combining drug and surgical intervention may be the key point to achieve the best health outcomes.

## Challenges to MetS in children and adolescents

A lack of awareness of MetS remains the biggest challenge for the management of MetS in children and adolescents. In a meta-analysis, 50.7% of parents underestimated the weight of their overweight/obese children (158). In fact, a chubby infant or child is often seen as a sign of good health and care in developing countries experience long periods of economic underdevelopment and material scarcity. The belief that “chubby kids are healthy” leads to delayed diagnosis and treatment of obesity in children. Fortunately, more and more attention has been paid to childhood obesity that has been shown to be linked to the development of MetS and CVD in adulthood (159). Autopsy studies have revealed that multiple cardiovascular risk factors are associated with early stages of coronary atherosclerosis (160). We assume that a high incidence of MetS among overweight adolescents, coupled with the rising prevalence of childhood obesity, could lead to a disproportionate increase in CVD in adulthood.

Diagnostic thresholds, whether based on percentiles or absolute numbers, need to be established based on objective disease endpoints to be meaningful. Moreover, these thresholds may need to be adjusted according to age or pubertal stage as children grow. Given the differing risk profiles across various ethnic groups, it is unclear whether the same standards should apply to different racial groups. Thus, any pediatric definition of MetS must be rigorously evaluated, which is a complex and challenging medical issue (161).

## Summary

The diagnosis and treatment of MetS is still a challenge in children and adolescents. Standardized diagnostic criteria and treatment protocols are urgently required to guide clinical practice. The prevalence, diagnosis, and treatment of MetS show a huge variance due to differences in economy among different countries and populations. The ideal treatment approach involves a collaborative effort between families, schools, and society. We should focus on improving dietary habits, increasing physical activity, reducing sedentary behavior, and enhancing energy expenditure in children. Given the complexity of MetS in children and adolescents, a multidisciplinary and multi-sectoral approach is necessary.
